# Molecular Characterization and Expression Analysis of *CD22* in Nile Tilapia (*Oreochromis niloticus*) and Its Potential Role in Immune Responses

**DOI:** 10.3390/biology15020140

**Published:** 2026-01-13

**Authors:** Qi Ye, Jimin Niu, Yu Huang, Jichang Jian

**Affiliations:** 1 Guangdong Provincial Key Laboratory of Aquatic Animal Disease Control and Healthy Culture & Key Laboratory of Control for Disease of Aquatic Animals of Guangdong Higher Education Institutes, Fisheries College, Guangdong Ocean University, Zhanjiang 524088, China; yeqi2002888@163.com (Q.Y.); 18378718901@163.com (J.N.); 2 Guangdong Provincial Engineering Research Center for Aquatic Animal Health Assessment, Shenzhen 327005, China

**Keywords:** Nile tilapia, *CD22*, immune regulation, signal pathway

## Abstract

In mammals, *CD22* is a member of the Siglec family and plays essential roles in B-cell activation, signal transduction, and immune regulation. However, its functions in teleost fish remain largely unclear. In this study, a *CD22* homolog designated *On-CD22* was identified and cloned from Nile tilapia (*Oreochromis niloticus*). *On-CD22* transcripts were highly expressed in the head kidney and peripheral blood of healthy fish and showed significant expression changes following infection with *Streptococcus agalactiae*, *Aeromonas hydrophila*, or stimulation with poly(I:C). Subcellular localization analysis indicated that *On-CD22* is predominantly localized to the plasma membrane. Luciferase reporter assays performed in heterologous cell systems showed that overexpression of *On-CD22* was associated with changes in the basal transcriptional activities of NF-κB, IFN1, IFN3, and STAT1 responsive promoters under unstimulated conditions. Furthermore, single-cell transcriptomic analysis revealed that *On-CD22* expression was mainly confined to the B-cell population within head-kidney leukocytes. Collectively, these findings suggest that *On-CD22* may be involved in immune regulatory processes in Nile tilapia.

## Simple Summary

*CD22* (*Siglec-2*) is an inhibitory co-receptor on mammalian B cells that plays an important role in immune regulation, whereas its functions in teleost fish are poorly understood. In this study, a *CD22* homolog (*On-CD22*) was identified in Nile tilapia and examined under immune challenge conditions. *On-CD22* was highly expressed in immune-related tissues and exhibited dynamic expression changes following bacterial infection or viral mimic stimulation. The protein was localized to the plasma membrane, and overexpression assays were associated with alterations in the basal activities of NF-κB, interferon, and STAT1 related signaling reporters. These findings suggest that *On-CD22* may be involved in immune regulatory processes in Nile tilapia.

## 1. Introduction

The immunoglobulin superfamily (IgSF) comprises a diverse group of cell-surface molecules that mediate cell–cell and cell–matrix adhesion through the specific recognition of glycoproteins and sialylated glycans [1]. These interactions participate in a wide range of biological processes, including immune activation, apoptosis, inflammatory regulation, and control of cell proliferation [2]. Among the IgSF members, sialic acid–binding immunoglobulin-like lectins (Siglecs) are predominantly expressed on immune and hematopoietic cells and exhibit distinct cell-type-specific expression patterns. Upon engagement with sialylated glycan ligands, Siglecs can transmit inhibitory signals via their intracellular domains, thereby modulating immune cell activation and maintaining immune homeostasis [3]. Structurally, Siglecs are type I transmembrane receptors characterized by an N-terminal V-set immunoglobulin domain responsible for sialic acid recognition, followed by one to sixteen C2-set Ig-like domains positioned proximal to the cell membrane [4,5]. Based on phylogenetic relationships, the Siglec family is classified into two major clades: evolutionarily conserved Siglecs (including CD169/Siglec-1, CD22/Siglec-2, MAG/Siglec-4, and Siglec-15) and the rapidly diversified CD33-related Siglecs that are largely restricted to mammals [6,7]. Through recruitment of intracellular phosphatases and modulation of downstream signaling pathways, Siglecs contribute to the regulation of inflammatory responses, leukocyte homeostasis, host–pathogen interactions, and immune surveillance in cancer [8,9,10].

*CD22* (also known as *Siglec-2*) is one of the most extensively characterized members of the Siglec family. It was initially identified as a B-lymphocyte-specific transmembrane glycoprotein by Wilson et al. (1991), who cloned its full-length cDNA and designated it B-lymphocyte cell adhesion molecule (BL-CAM) [11]. *CD22* is a type I transmembrane glycoprotein of approximately 140 kDa, with a cytoplasmic tail containing immunoreceptor tyrosine-based inhibitory motifs (ITIMs) that are essential for its signaling function [12,13]. Its extracellular region consists of an N-terminal V-set Ig domain followed by multiple C2-set Ig-like domains. In mammals, *CD22* is primarily expressed on B lymphocytes and functions as a key inhibitory co-receptor that maintains B-cell homeostasis by limiting excessive activation of the B-cell receptor (BCR) [14,15,16]. Upon BCR engagement, tyrosine residues within *CD22* ITIMs become phosphorylated, enabling the recruitment of the phosphatases SHP1 and SHP2 and subsequent attenuation of downstream signaling cascades [17,18]. In addition, *CD22* has been reported to modulate Toll-like receptor 7–mediated B-cell responses by influencing NF-κB activation and pro-survival signaling pathways [19] and to interact with other membrane proteins such as β7 integrins, thereby affecting B-cell homing and migration [20]. Dysregulation or aberrant expression of *CD22* is associated with excessive B-cell activation and has been implicated in autoimmune diseases and B-cell malignancies, including diffuse large B-cell lymphoma (DLBCL) [21,22].

In teleost fish, the head kidney serves as a major hematopoietic and immune organ analogous to mammalian bone marrow, supporting the development and differentiation of multiple leukocyte lineages collectively referred to as head-kidney leukocytes (HKLs) [23]. With the increasing application of bulk and single-cell transcriptomic approaches, HKLs have been shown to comprise several major immune cell subsets, providing a valuable model for investigating immune gene regulation and cellular responses during pathogen challenge. Despite the well-established roles of *CD22* in mammalian immunity, functional studies of *CD22* in teleost fish remain limited but have begun to emerge in recent years. Li et al. reported that the incubation of peripheral blood leukocytes from *Cynoglossus semilaevis* with an anti-*CD22* antibody enhanced cell proliferation, phagocytosis, and antibacterial activity, suggesting an inhibitory role of *CD22* in leukocyte activation [24]. In *Japanese flounder*, *PoCD22* was broadly expressed in IgM^+^ B cells and regulated phagocytosis and reactive oxygen species (ROS) production in a tissue-dependent manner, indicating that *CD22* may participate in the regulation of B-cell-associated innate immune functions in teleosts [25]. In Nile tilapia, although *CD22* itself has not been systematically characterized, several Siglec family members, including *Siglec1-like*, *Siglec4-like*, *Siglec7*, and *Siglec14-like*, have been implicated in host responses to *Streptococcus agalactiae* infection [26,27]. Together, these studies support the idea that Siglec-mediated inhibitory regulation is conserved in teleost immunity, while the specific expression features and functional relevance of *CD22* in tilapia remain largely unexplored.

*Oreochromis niloticus* (Nile tilapia) is one of the most important aquaculture species worldwide, with China representing a major production region [28,29]. Although tilapia exhibit strong environmental adaptability, intensive aquaculture practices have led to frequent outbreaks of infectious diseases caused by pathogens such as *Streptococcus agalactiae* and *Aeromonas hydrophila*, resulting in high mortality rates and substantial economic losses [30]. Therefore, elucidating the immune mechanisms underlying pathogen resistance in tilapia, particularly regulators bridging innate and adaptive immunity, is essential for developing targeted disease-control strategies. Here, we identified a *CD22* homolog (On-CD22) from *O. niloticus* and profiled its molecular features, tissue distribution, and transcriptional responses to bacterial infection and poly(I:C) stimulation. By combining immune challenge-associated expression analyses with head-kidney leukocyte transcriptomic evidence and gain-of-function reporter assays, this study places teleost *CD22* within cellular and immunological context and provides a basis for future mechanistic and in vivo functional investigations.

## 2. Materials and Methods

### 2.1. Animals

Healthy Nile tilapia (*Oreochromis niloticus*) with an average body weight of 50 ± 10 g were obtained from the Zhanjiang Tilapia Breeding Facility (Zhanjiang, China). Fish were acclimated for 28 days in 1000 L aerated freshwater tanks under controlled conditions (stocking density: 4 g/L; temperature: 28 ± 2 °C) and were fed a commercial diet at 3% of body weight per day [31]. All procedures complied with the Guangdong Provincial Laboratory Animal Management Guidelines [32].

### 2.2. Pathogenic Bacteria and Poly(I:C) Challenge

The *Streptococcus agalactiae* strain ZQ1901, which was originally isolated from a diseased tilapia and held in our laboratory collection [33]. The bacteria were grown overnight at 28 °C in Brain Heart Infusion (BHI) broth. After an 18 h incubation, we harvested the cells by centrifugation (4000× *g*, 5 min), resuspended in sterile PBS to a final concentration of 5 × 10^7^ CFU/mL. *Aeromonas hydrophila*, another fish pathogen from our collection, was cultured using the same procedure, culturing it in LB broth under identical conditions. For the viral mimic, we prepared a solution of poly(I:C) in sterile PBS at 1 mg/mL and stored at −20 °C.

A total of 150 healthy Nile tilapia were randomly divided into three groups (*n* = 50 per group): the *Streptococcus agalactiae*-challenged group, *Aeromonas hydrophila*-challenged group, and poly(I:C)-treated group. Fish in the bacterial challenge groups were intraperitoneally (i.p.) injected with 0.1 mL per fish of *S. agalactiae* (5 × 10^7^ CFU/mL) or *A. hydrophila* (5 × 10^7^ CFU/mL). Fish in the poly(I:C) group were intraperitoneally injected with 0.1 mL of poly(I:C) (1 mg/mL), corresponding to 0.1 mg per fish (approximately 2 mg/kg body weight). At each time point, three fish from each group were randomly selected and euthanized as independent biological replicates (*n* = 3). Tissue samples (spleen, brain, head kidney, liver, and intestine) were collected at 0, 6, 12, 24, and 48 h post-injection, snap-frozen in liquid nitrogen, and stored at −80 °C for RNA extraction. No PBS-injected sham control was included and the 0 h samples prior to challenge were used as baseline controls.

### 2.3. RNA Extraction cDNA Synthesis

Total RNA was isolated from tissue samples based on a previously described method [34]. Briefly, following the manufacturer’s guidelines (TransGen, Beijing, China), samples were homogenized in TransZol Up Plus reagent for total RNA isolation. Subsequent quantification of the extracted RNA was performed using a NanoDrop 2000 system (Thermo Fisher Scientific, Waltham, MA, USA). Reverse transcription to generate first-strand cDNA was carried out with the TransScript One-Step SuperMix Kit (TransGen, Beijing, China), which integrates genomic DNA removal before the cDNA synthesis reaction. The final cDNA products were aliquoted after a 1:50 dilution with nuclease-free water and maintained at −20 °C for future quantitative real-time PCR experiments [35].

### 2.4. Quantitative Real-Time PCR (qRT-PCR)

The expression profiles of On-CD22 were examined in five tissues from healthy and challenged Nile tilapia. Total RNA was extracted and reverse-transcribed as described above. Quantitative real-time PCR (qRT-PCR) was performed on an Applied Biosystems 7500 Real-Time PCR System (Thermo Fisher Scientific, Waltham, MA, USA) using SYBR Green chemistry. Each 10 μL reaction contained 5 μL of 2× Takara Ex Taq™ SYBR Premix, 0.5 μL of diluted cDNA, 0.5 μL each of forward and reverse primers, and 3.5 μL of nuclease-free water. The cycling program consisted of 94 °C for 20 s, followed by 40 cycles of 94 °C for 20 s, 52 °C for 10 s, and 72 °C for 15 s. Melting curve analysis was performed at the end of each run to confirm amplification specificity. Each sample was run in triplicate technical replicates. β-Actin and GAPDH were used as reference genes for normalization, and their stability was assessed by examining Ct variation across treatments and time points, with no obvious systematic shifts observed. Primer specificity was confirmed by melting curve analysis, and all primer pairs yielded a single peak. Relative expression levels were calculated according to the normalization strategy described by Vandesompele and Hellemans [36,37], using the 2^−ΔΔCt^ method under the assumption of comparable amplification efficiencies.

### 2.5. Cloning and Bioinformatics Analysis of Nile Tilapia On-CD22 Gene

Based on the *CD22* gene sequence available in NCBI (Nile tilapia, *Oreochromis niloticus* LOC102077587), specific primers for *On-CD22* (Table 1) were designed using Primer 5.0, with head kidney cDNA as a template. The following primers were used in the amplification reactions (20 μL volume): 10 μL Premix Taq™ PCR Master Mix, 7.5 μL nuclease-free H_2_O, 0.5 μL cDNA template, and 1.0 μL for each gene-specific primer (forward/reverse) with the thermal cycling parameters: Initial denaturation: 94 °C × 5 min; 34 cycles of [94 °C × 30 s →60 °C × 30 s→72 °C × 60 s]; Final extension: 72 °C × 10 min; Terminal hold: 4 °C.

Multi-sequence alignments were executed via the NCBI BLAST (https://www.ncbi.nlm.nih.gov/; accessed on 10 November 2025); signal peptide profiling employed SignalP 5.0 (http://www.cbs.dtu.dk/services/SignalP/; accessed on 10 November 2025); genome structure analysis with Exon-Intron Graphic Maker; transmembrane structure analysis via TMHMM-2.0 (http://www.cbs.dtu.dk/services/TMHMM/; accessed on 10 November 2025); amino acid composition analysis using ExPASy ProtParam (http://web.expasy.org/protparam/; accessed on 10 November 2025) and amino acid site characterization with SoftBerry (http://linux1.softberry.com; accessed on 10 November 2025); protein conserved domain prediction was carried out using SMART (http://smart.embl-heidelberg.de/smart/; accessed on 10 November 2025); for structural analyses, DNAMAN 9.0 and PyMOL 3.1 were employed for sequence alignment. The method of neighbor-joining (NJ) with 1000 bootstrap replicates was used to establish the phylogenetic trees by MEGA7.0 [38,39].

### 2.6. Subcellular Localization Assay

GS cells (a grouper spleen–derived cell line) were cultured at 28 °C in Leibovitz’s L-15 medium (Gibco, Waltham, MA, USA) containing 10% fetal bovine serum (FBS). To investigate subcellular localization, the cells were plated on glass coverslips placed in 24-well plates. Transfection was performed with either the *pEGFP-OnCD22* plasmid or the empty *pEGFP-C1* vector as a control, using Lipofectamine^®^ 3000 (Thermo Fisher Scientific, Waltham, MA, USA) according to the manufacturer’s instructions. Following a 24 h incubation, cells with rinsed PBS and fixed for 10 min using 4% paraformaldehyde. Nuclei were stained with 1 µg/mL DAPI (Beyotime Biotechnology, Shanghai, China). A confocal laser scanning microscope (Zeiss Axioscope5, Jena, Germany) was used to capture fluorescence images and determine the localization of the *On-CD22* fusion protein within the cells.

### 2.7. Plasmid Construction

The plasmid construction was carried out following the protocol outlined by Huang et al. [40]. Two recombinant plasmids, *pcDNA3.1-CD22* and *pEGFP-OnCD22*, were constructed for functional and subcellular localization studies, respectively.

For construction of *pcDNA3.1-CD22*, gene-specific primers containing BamHI and XhoI restriction sites were designed according to the full-length coding sequence (CDS) of *On-CD22*. The CDS fragment (without the stop codon) was amplified from Nile tilapia head-kidney cDNA using a high-fidelity DNA polymerase (TransGen, Beijing, China). The purified PCR product and the pcDNA3.1 vector were digested with BamHI/XhoI, gel-purified, and ligated using T4 DNA ligase (TaKaRa, Kusatsu, Shiga, Japan) to generate a construct encoding His-tagged *On-CD22*. For *pEGFP-OnCD22*, the same CDS fragment was cloned into the BamHI/XhoI sites of *pEGFP-N1* (Clontech, Mountain View, CA, USA) to obtain a C-terminal EGFP fusion plasmid. Recombinant plasmids were transformed into *E. coli* DH5α competent cells and verified by Sanger sequencing. Endotoxin-free plasmid DNA was prepared using the E.Z.N.A.^®^ Endo-Free Plasmid Mini Kit (Promega, Madison, WI, USA) and stored at −20 °C until use. Expression of *pcDNA3.1-CD22* was confirmed by Western blotting using a mouse anti-His tag primary antibody and a goat anti-mouse secondary antibody.

### 2.8. Transfection and Luciferase Assay

HEK293T and FHM cells were plated in 24-well plates and allowed to grow until they reached approximately 70–80% confluence at the time of transfection. To avoid interference with the formation of lipid–DNA complexes, antibiotic-free medium supplemented with 10% FBS was used during the procedure. For each well, a plasmid mixture was prepared containing 125 ng of the Firefly reporter construct (*pNF-κB-luc*, *pIFN1-luc*, *pIFN3-luc*, or *pSTAT1-luc*), 4 ng of the Renilla control plasmid *pRL-TK*, and 125 ng of *pcDNA3.1-CD22*. The control cells were supplemented with an equivalent amount of empty vector. For the transfection mix, the plasmid DNA was first blended with 0.5 μL of P3000 reagent, and the volume was adjusted to 25 μL with Opti-MEM. Separately, 1 μL of Lipofectamine^®^ 3000 was diluted in 25 μL of Opti-MEM. The two solutions were then combined, mixed gently, and kept at room temperature for about 10–15 min to allow the complexes to assemble. The resulting mixture was dispensed dropwise into each well, which contained 450 μL of fresh medium. No additional immune stimuli (such as TNF-α, poly(I:C), LPS, or bacterial components) were applied during the luciferase assays.

Roughly 24 h later, Firefly and Renilla activities were quantified using the Dual-Luciferase^®^ Reporter Assay System (Promega, Madison, WI, USA). Relative promoter activity was determined by dividing Firefly luminescence by the Renilla internal control (RLU_1_/RLU_2_). All assays included three independent biological samples, with each sample analyzed in triplicate technical wells.

### 2.9. Single-Cell Transcriptome Analysis of Tilapia HKLs

Single-cell RNA-Seq data of Nile tilapia head-kidney leukocytes were obtained from a previously published dataset [41] (accession number: PRJNA1304741&PRJNA1304859, https://www.ncbi.nlm.nih.gov/bioproject/ PRJNA1304741 and https://www.ncbi.nlm.nih.gov/bioproject/PRJNA1304859 (accessed on 10 November 2025)) generated using 10× Genomics. Raw count matrices were processed using standard Seurat workflows, including quality filtering (cells with low gene counts or high mitochondrial gene proportions were excluded), normalization, principal component analysis, and graph-based clustering. Cell types were annotated based on canonical immune marker genes as previously described. HKLs were resolved into five major immune cell populations. In this study, the expression pattern of *On-CD22* was re-examined using the online analysis platform Omicsmart (https://demo.omicsmart.com/10X/home.html#/ (accessed on 10 November 2025)). The transcript distribution across HKL subsets was first visualized using t-SNE, which provided a nonlinear dimensionality reduction and clustering overview of gene expression. Subsequently, the relative expression intensity of *On-CD22* was compared among the identified lymphoid and myeloid cell subgroups. In addition, transcriptome data of Nile tilapia HKLs collected 24 h after infection with *Streptococcus agalactiae* or *Aeromonas hydrophila* were examined. These datasets were used to quantify the expression levels of *On-CD22* across different samples and to assess its differential expression under the two bacterial challenge conditions.

### 2.10. Data Visualization and Statistical Analysis

Data are presented throughout as mean ± SD. The software packages GraphPad Prism (v8.0), Adobe Illustrator 2021 and Adobe Photoshop CC 2019 (https://js.design/special/ps/, accessed on 10 November 2025) were utilized for statistical analysis and figure preparation, respectively. Significance testing involved either the Student’s *t*-test or one-way ANOVA with Tukey’s post hoc test, with a *p* < 0.05 deemed significant. The figures themselves denote significant differences using asterisks or distinct letters [42].

## 3. Results

### 3.1. Bioinformatics Analysis of Nile Tilapia On-CD22 ORF Sequence

**Figure 1:**
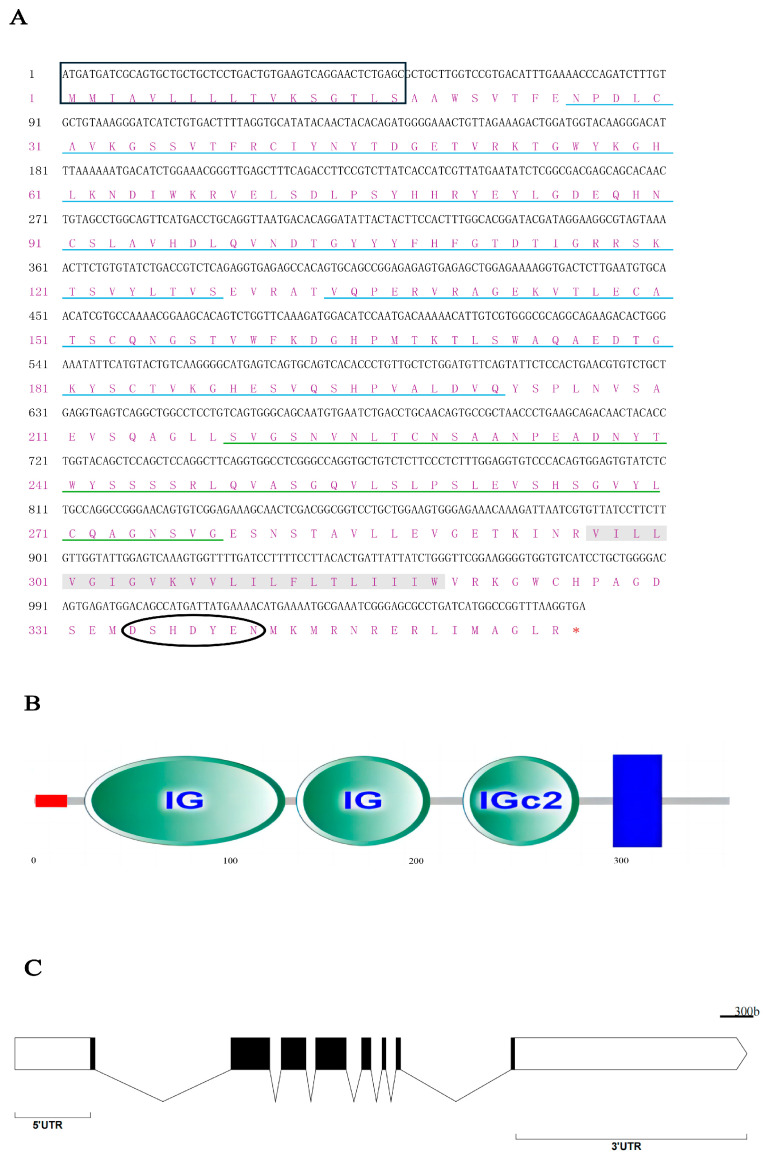
(**A**) The sequence and structural characteristics of *CD22* in tilapia. (**A**) The sequence analysis of *Oreochromis niloticus CD22* (Siglec2), the black box is the signal peptide region, and the blue underlined area is the IG structural domain, the green underlined area is the IGc2 structural domain, the gray shaded boxes are transmembrane structural domains, black circles indicate ITSM-like motif, and “*” is the stop codon; (**B**) The conserved domain prediction of *On-CD22*; (**C**) Genomic structure of *On-CD22*, Black boxes represent exons.

**Figure 2:**
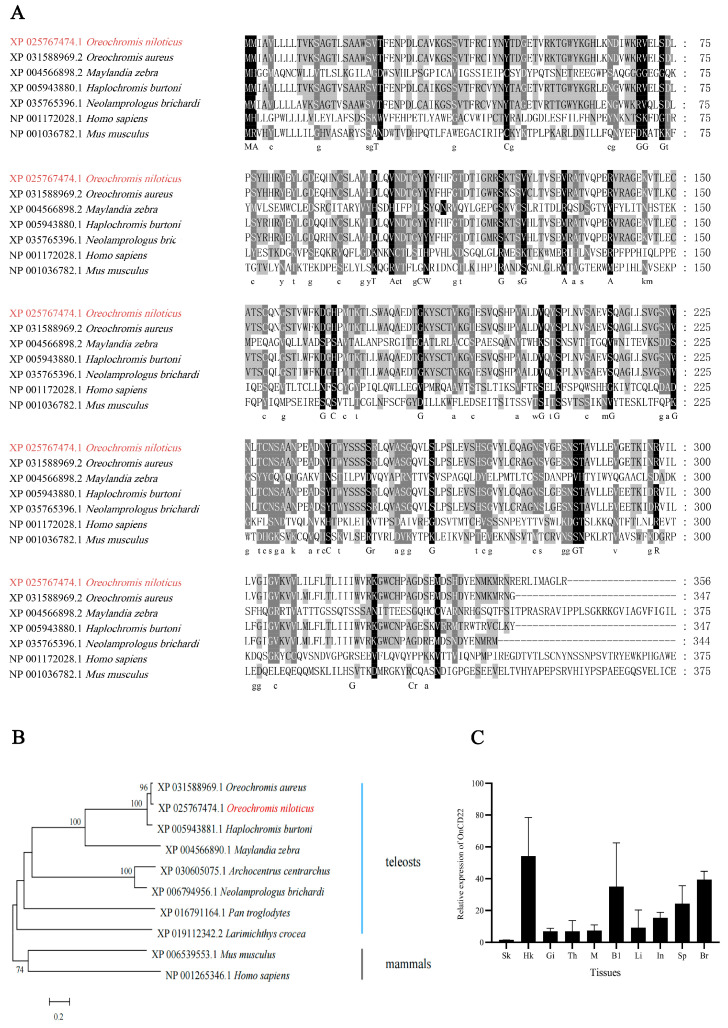
(**A**) Cross-species amino acid sequence conservation analysis of *Oreochromis niloticus CD22*. Residues with a black background indicate highly conserved amino acids across species; residues with a gray background indicate conserved substitutions; unshaded positions indicate low conservation. (**B**) Phylogenetic reconstruction of *On-CD22* orthologs across diverse taxa. (**C**) *CD22* mRNA level in different tissues in tilapia as determined by qRT-PCR. Sk: Skin; Hk: Head Kidney; Gi: Gill; Th: Thymus; M: Muscle; Bl: Blood; Li: Liver; In: Intestine; Sp: Spleen; Br: Brain. Each data point represents the mean ± standard deviation (SD); *n* = 3 (*n*: each analyzed in triplicate).

*On-CD22* ORF 1071bp encodes a protein of 356 aa, and the amino acid residues are indicated in Figure 1A, which shows the signal peptide from amino acid positions 1 to 18, with a cleavage site between positions 18 and 19, and an ITSM-like inhibitory motif at 335–341 (Figure 1A). The theoretical molecular formula of *On-CD22* is C_1736_H_2722_N_480_O_533_S_16_, which corresponds to a molecular mass of approximately 39.35 kDa and an isoelectric point (pI) of 6.46. Predicted structural analysis indicates that *On-CD22* is a type I transmembrane glycoprotein, comprising two immunoglobulin variable (IgV) domains (residues 26–128 and 134–202) and one immunoglobulin constant (IgC2) domain (residues 219–278) connected by a short coiled-coil linker (Figure 1B). Genomic analysis showed that *On-CD22* comprises eight exons and seven introns, forming a genomic structure that is conserved among vertebrate *CD22* homologs (Figure 1C). Phylogenetic analysis based on amino acid sequences of *CD22* orthologs from representative species showed that Nile tilapia *CD22* grouped most closely with *Oreochromis aureus* (96.53% sequence identity), while displaying distant relationships with mammalian counterparts, including *Mus musculus* (32.5%) and *Homo sapiens* (31.07%) (Figure 2A,B). Collectively, these findings indicate that *On-CD22* is a conserved teleost homolog of mammalian *CD22*, retaining the characteristic immunoglobulin architecture typical of Siglec-2 family members.

### 3.2. Tissue Distribution of CD22 Healthy Tilapia

qRT-PCR was performed to examine the relative expression levels of *On-CD22* in various tissues of in healthy Nile tilapia. The transcripts were most abundant in the head kidney and peripheral blood, whereas the skin displayed the lowest expression (Figure 2C).

### 3.3. The Transcriptional Expression of On-CD22 After Infected Tilapia

**Figure 3:**
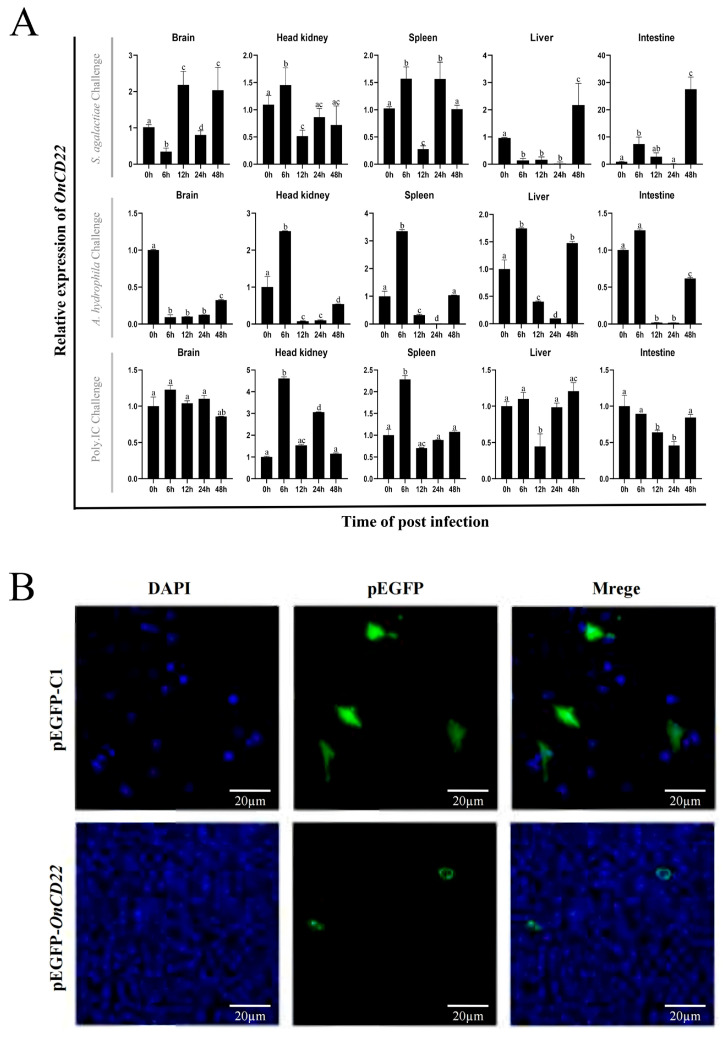
(**A**) Relative expression of *On-CD22* in five tissues after infection with *S. agalactiae*, *A. hydrophila*, and *Poly(I:C)*. The expression level at 0 h was normalized to 1.00 to calculate the relative expression at later time points. Data are shown as mean ± SD, *n* = 3 (*n*: each analyzed in triplicate). Statistically significant differences are indicated by different letters (*p* < 0.05); (**B**) The subcellular localization of *On-CD22* in GS cell.

Following immune challenge, a significant upregulation (*p* < 0.05) in the transcriptional levels of *On-CD22* was observed across all tested tissues—brain, head kidney, intestine, liver, and spleen. The expression dynamics, however, varied with the type of pathogen. Upon *Streptococcus agalactiae* infection, the brain, head kidney, and spleen exhibited a transient increase in *On-CD22* expression between 6 and 24 h post-infection (hpi), which returned to baseline by 48 hpi. In contrast, infection with *Aeromonas hydrophila* induced a sharp, short-term response in the head kidney and spleen, peaking at approximately 3–3.5-fold at 6 hpi before a rapid decline. When stimulated with the viral mimic Poly(I:C), the head kidney showed a particularly strong and sustained induction, with expression rising markedly (around fivefold) by 6 h and remaining elevated at 24 h (Figure 3A).

### 3.4. Subcellular Localization of On-CD22

GS cells were transfected with *pEGFP-OnCD22* to analyze its subcellular localization. As shown in Figure 3B, *pEGFP-OnCD22* fusion protein localized predominantly to the plasma membrane, while cells transfected with the empty vector *pEGFP-N1* showed diffuse cytoplasmic and nuclear fluorescence. DAPI staining confirmed that *On-CD22* was excluded from the nucleus, supporting its membrane association.

### 3.5. Effect of On-CD22 Overexpression on Basal NF-κB Promoter Activity

**Figure 4:**
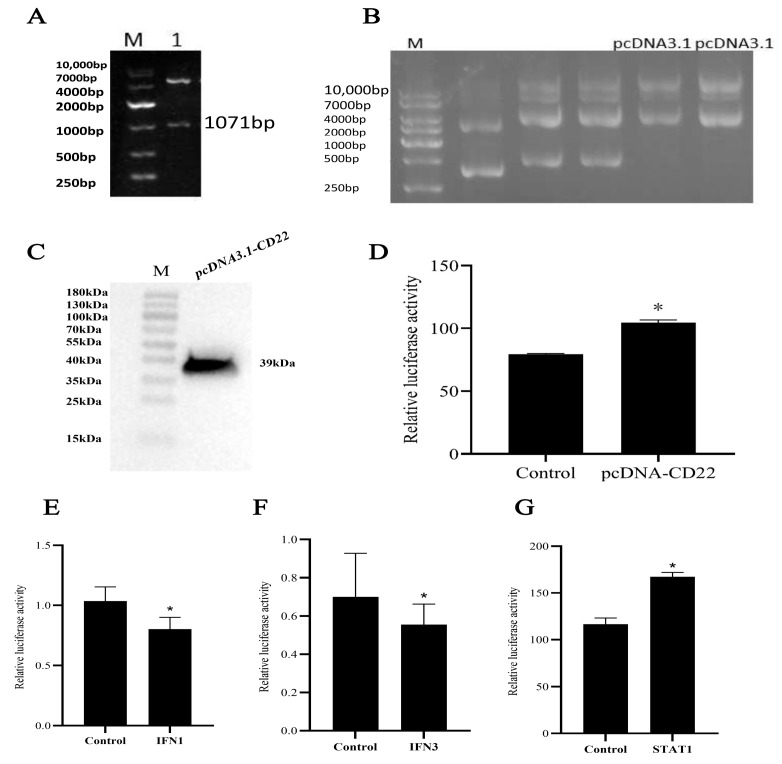
(**A**) Enzyme digestion results of recombinant plasmid *pcDNA-CD22*. M: DNA Molecular Marker DL10,000; 1: target gene; (**B**) Empty *pcDNA3.1* plasmid extraction results. M: DNA Molecular Marker DL10,000; (**C**) Analysis of *pcDNA3.1-CD22* expression in HEK293T cells with Western blot. M: Protein Molecular Marker; the original uncropped Western blot figure in Supplementary Materials Figure S1; (**D**–**G**) Effect of *On-CD22* overexpression on NF-κB activity in HEK293T cells; effect of *IFN1*, *IFN3*, and *STAT1* on *On-CD22* in FHM cells. Data expressed as the relative luciferase activities. “*”: *p* < 0.05 compared with the control group. The significant difference analysis was performed via Student’s *t*-tests and indicated with asterisks (*p* < 0.05).

Overexpressed *On-CD22* in 293T cells together with a *pRL-TK* plasmid to test its effect on the NF-κB pathway. Prior to this functional assay, successful expression of the *On-CD22* protein in this system was confirmed by Western blot, which detected a band corresponding to the anticipated molecular weight (Figure 4C). The results show that *On-CD22* significantly altered the basal activity of the NF-κB-responsive luciferase reporter under unstimulated conditions (Figure 4D).

### 3.6. Effects of On-CD22 on IFN1, IFN3, and STAT1

To investigate how *On-CD22* influences interferon-related signaling and the transcriptional activity of *STAT1*, dual-luciferase reporter assays were performed in FHM cells. The results show that under unstimulated conditions, *On-CD22* overexpression significantly reduced the basal activities of IFN1 and IFN3 promoters, while a modest but significant increase in basal STAT1 promoter activity was observed (Figure 4E–G).

### 3.7. Expression Characteristics of On-CD22 in HKL scRNA-Seq

**Figure 5:**
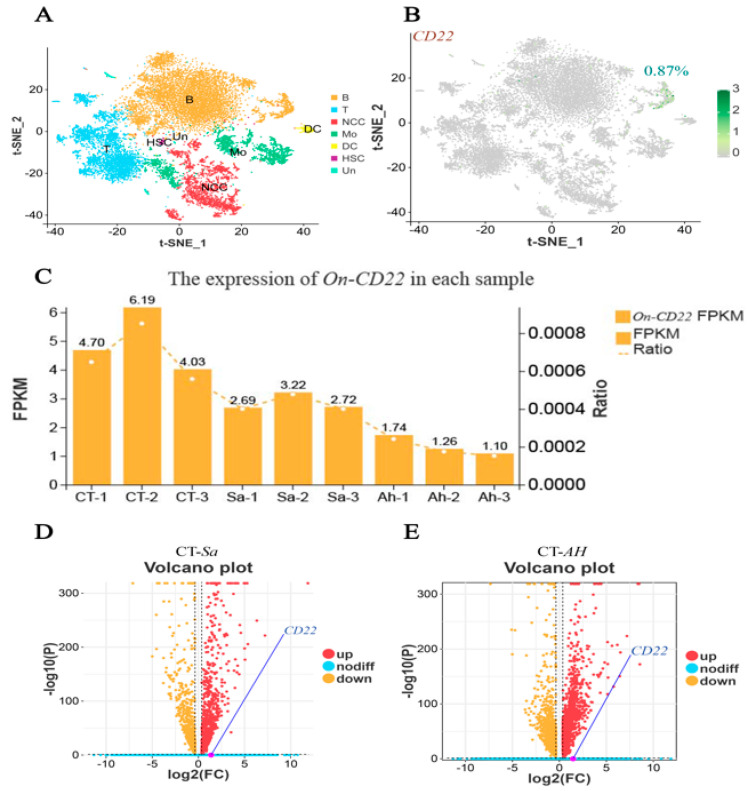
(**A**) t-SNE visualization of single-cell transcriptomic data showing major immune cell populations in Nile tilapia; (**B**) Expression distribution of *CD22*; (**C**) Bar plots show the FPKM values of *On-CD22* in each sample (left *y*-axis). CT indicates healthy controls, Sa indicates *Streptococcus agalactiae*–challenged samples, and Ah indicates *Aeromonas hydrophila*–challenged samples. The dashed line shows the relative ratio of *On-CD22* expression (right *y*-axis); (**D**,**E**) Volcano plots showing differentially expressed genes under two pathogen-challenge conditions.

Single-cell transcriptomic analysis of head-kidney leukocytes (HKLs) identified five major immune cell subsets, including hematopoietic stem cells (HSCs), B lymphocytes, T lymphocytes, nonspecific cytotoxic cells (NCCs), and macrophage/monocyte-like cells (Mo) (Figure 5A). Among all HKLs, *On-CD22* expression was predominantly detected in the B-cell cluster, with approximately 0.87% of total HKL cells showing positive expression (Figure 5B). *On-CD22* expression was detected in approximately 0.87% of total HKLs, corresponding to approximately 60 *CD22*-positive cells out of 6933 total cells. This proportion was comparable across biological replicates, indicating consistent expression patterns. Bulk RNA-Seq analysis of head-kidney leukocytes (HKLs) at 24 h post-infection showed that *On-CD22* expression was highest in the control group, with FPKM values ranging from 4.03 to 6.19. Following *Streptococcus agalactiae* challenge, *On-CD22* transcript levels decreased to 2.69–3.22, corresponding to an approximately 40–50% reduction relative to controls. A more pronounced decrease was observed in the *Aeromonas hydrophila*–infected group, with FPKM values further reduced to 1.10–1.74 (Figure 5C). Although these reductions indicate a consistent downward expression trend, volcano plot analysis showed that the magnitude of change did not reach the predefined thresholds for differential expression in the global RNA-Seq analysis (Figure 5D,E). As a result, *On-CD22* was positioned near the non-significant region of the volcano plots. This reflects the relatively modest overall transcriptional alterations in HKLs at 24 h post-infection, where only a limited subset of genes met the criteria for differential expression.

## 4. Discussion

Siglec family receptors are widely recognized as immunomodulatory lectins that fine-tune immune cell activation through the recognition of sialic acid-containing ligands and the engagement of intracellular inhibitory motifs. Through these mechanisms, Siglecs contribute to the regulation of inflammatory responses, leukocyte homeostasis, host–pathogen interactions, and immune surveillance in cancer [43,44]. *CD22* is the best-characterized member of the Siglec family [45], and its immunoregulatory functions have been extensively studied in mammals [46,47,48]. In contrast, in teleost fish, the structural features, expression patterns, and functional implications of *CD22* remain incompletely understood. In the present study, we integrated molecular characterization, immune challenge-associated expression analysis, head-kidney leukocyte transcriptomic data, and gain-of-function reporter assays to investigate *CD22* in Nile tilapia.

We characterized the molecular features and immune-related functions of *On-CD22* in Nile tilapia. Sequence analysis showed that *On-CD22* contains an N-terminal signal peptide, two Ig domains, an IgC2 domain, and a transmembrane region. This domain architecture is consistent with Siglec homologs reported in other teleost species, indicating evolutionary conservation [25]. Phylogenetic analysis showed that *On-CD22* clusters with other teleost *CD22* orthologs and is clearly separated from mammalian counterparts.

qRT-PCR analysis revealed widespread *On-CD22* expression in healthy Nile tilapia tissues, with particularly high levels in the head kidney and peripheral blood—both key hematopoietic sites. As the head kidney functionally parallels mammalian bone marrow and supports immune cell development [49,50], the prominent expression of *On-CD22* there, along with its detection in peripheral blood leukocytes, strongly suggests its involvement in immune surveillance. This observation is consistent with established patterns of Siglec expression in teleost immune tissues such as the spleen and kidney [26,27]. A comparative study of four Siglec family members (Siglec-1, *CD22*, MAG, and Siglec-15) in teleost also reported high expression in the spleen, head kidney and gill [51]. Following bacterial (*Streptococcus agalactiae* and *Aeromonas hydrophila*) and viral mimic (Poly: IC) challenges, *On-CD22* expression was upregulated across multiple tissues, especially in the head kidney and spleen. The rapid induction observed within the first 6–24 h post-infection corresponds to early innate immune activation and is consistent with the dynamic expression of other immune genes such as *IL-1β* and *TLR5* in tilapia [52,53]. Together, these observations suggest that *On-CD22* may be involved in early immune regulatory processes following infection, potentially contributing to the modulation of immune activation through its intracellular inhibitory motifs. Subcellular localization analysis confirmed that *On-CD22* is localized to the cell membrane, supporting its predicted function as a type I cell surface receptor.

The functional assays provide insight into the potential immunoregulatory properties of *On-CD22*. In heterologous cell systems, *On-CD22* overexpression was associated with alterations in the basal promoter activities of NF-κB, IFN1, IFN3, and STAT1 responsive reporters under unstimulated conditions. Specifically, increased basal activities of NF-κB and STAT1 reporters were observed, whereas the basal activities of IFN1 and IFN3 promoters were reduced. Rather than indicating a purely inhibitory or activating role, this pattern suggests that *On-CD22* may differentially modulate multiple immune-related transcriptional pathways in a context-dependent manner. In mammals, several Siglec family members have been shown to restrict type I interferon production as part of mechanisms that maintain immune homeostasis [54]. In the present study, the observation that *On-CD22* overexpression suppresses basal IFN1/IFN3 promoter activity is consistent with the conserved inhibitory role of *CD22* across vertebrates. However, the concurrent enhancement of NF-κB and STAT1 reporter activity suggests that teleost *CD22* may engage additional or context-dependent signaling outputs, potentially reflecting differences in immune cell composition, receptor crosstalk, or downstream signaling architecture between teleosts and mammals. Similar context-dependent signaling has been noted across the Siglec family, where downstream outputs can vary with cell type, activation state, and the availability/organization of cis- and trans-sialylated ligands [10]. Notably, the present study did not directly examine ITIM/ITSM tyrosine phosphorylation, phosphatase recruitment (e.g., SHP1/2), or signaling-deficient ITIM/ITSM mutant constructs. In addition, the reporter assays were conducted in heterologous cell lines rather than tilapia-derived primary immune cells, which may limit the physiological relevance of the observed signaling changes. Therefore, the precise molecular mechanisms and in vivo significance of these basal reporter alterations remain to be clarified in future studies.

Single-cell analysis revealed that *On-CD22* expression is mainly confined to the B-cell population, with approximately 0.87% of HKLs showing detectable transcript levels. The fraction of *On-CD22* positive HKLs was low, which is not unexpected given that *CD22* is primarily associated with B cells and may be confined to a subset or activation state of this population in teleosts [55]. In addition, droplet-based scRNA-Seq has well-recognized dropout, so the detectable-cell percentage likely underestimates the true expression prevalence. Both the bulk RNA-Seq analysis and the in vivo expression assays consistently showed that *On-CD22* is expressed at relatively high levels in healthy fish, but its transcription showed a relative decrease at 24 hpi with *Streptococcus agalactiae* or *Aeromonas hydrophila*. The volcano plot indicates that HKLs exhibit only modest global transcriptional alterations at 24 h post-infection, with relatively few genes showing strong differential expression. Importantly, the apparent discrepancy between the tissue-level qRT-PCR data and the HKL transcriptomic profiles likely reflects differences in biological scale and temporal resolution rather than contradictory regulation of *On-CD22*. qRT-PCR was performed on whole tissues across multiple time points and therefore captures integrated signals from heterogeneous cell populations, including changes in cellular composition after challenge, whereas the bulk RNA-Seq/scRNA-Seq analyses were restricted to isolated HKLs at a single time point (24 h) and applied stringent differential-expression thresholds. Accordingly, *On-CD22* showed a relative decrease in HKLs at 24 h that did not meet the DEG cutoff, explaining its position near the non-significant region in the volcano plots; thus, early whole-tissue upregulation may coexist with modest or reduced expression within HKLs at 24 h, and the overall HKL transcriptomic shift at this time point remained moderate in magnitude.

Together, these findings suggest that *On-CD22* may participate in early immune regulatory processes in Nile tilapia and that its expression is dynamically modulated during bacterial challenge. However, these conclusions should be interpreted with caution, as the functional evidence is primarily derived from correlative expression analyses and gain-of-function assays in heterologous cell systems. Further studies employing loss-of-function approaches and in vivo validation will be required to define the precise role and mechanisms of *On-CD22* in teleost immunity.

## 5. Conclusions

In conclusion, the present study provides molecular and expression-based evidence for the presence of a *CD22* homolog in Nile tilapia and characterizes its tissue distribution and transcriptional responses under immune challenge conditions. Combined with reporter assays conducted in heterologous cell systems, our findings offer initial functional clues suggesting that *On-CD22* may be involved in immune regulatory processes in teleost fish.

## Figures and Tables

**Table 1:** Primer sequences used in this study.

Primer	Nucleotide Sequence (5′-3′)
CD22-1F	ATGATGATCGCAGTGCTGCTG
CD22-1071R	TCACCTTAAACCGGCCATGAT
CD22-663F	TGGGCAGCAATGTGAATC
CD22-858R	CCGCCGTCGAGTTGCTTT
β-actin-F	AACAACCACACACCACACATTTC
β-actin-R	TGTCTCCTTCATCGTTCCAGTTT
M13	CGCCAGGGTTTTCCCAGTCACGAC
GAPDH-F	CCGTTACCGTGGTGAAGTGT
GAPDH-R	AACATTGGAGCATCGGGTGA
RV	GAGCGGATAACAATTTCACACAGGA
CD22-pcDNA-F	CGCGGATCCATGCATCATCATCATCATCAT ATGATCGCAGTGCTGCTGCT
CD22-pcDNA-R	CCGCTCGAGTCACCTTAAACCGGCCATGAT

## Data Availability

Data are contained within the article and Supplementary Materials.

## Supplementary Materials

The following supporting information can be downloaded at: https://www.mdpi.com/article/10.3390/biology15020140/s1, Figure S1: The original uncropped Western blot figures.
