# Supplementary figures and images for: Molecular Characterization and Expression Analysis of CD22 in Nile Tilapia (Oreochromis niloticus) and Its Potential Role in Immune Responses
Source: Biology (Basel). 2026 Jan 13;15(2):140. doi: 10.3390/biology15020140 (PMC12837193; doi:10.3390/biology15020140)

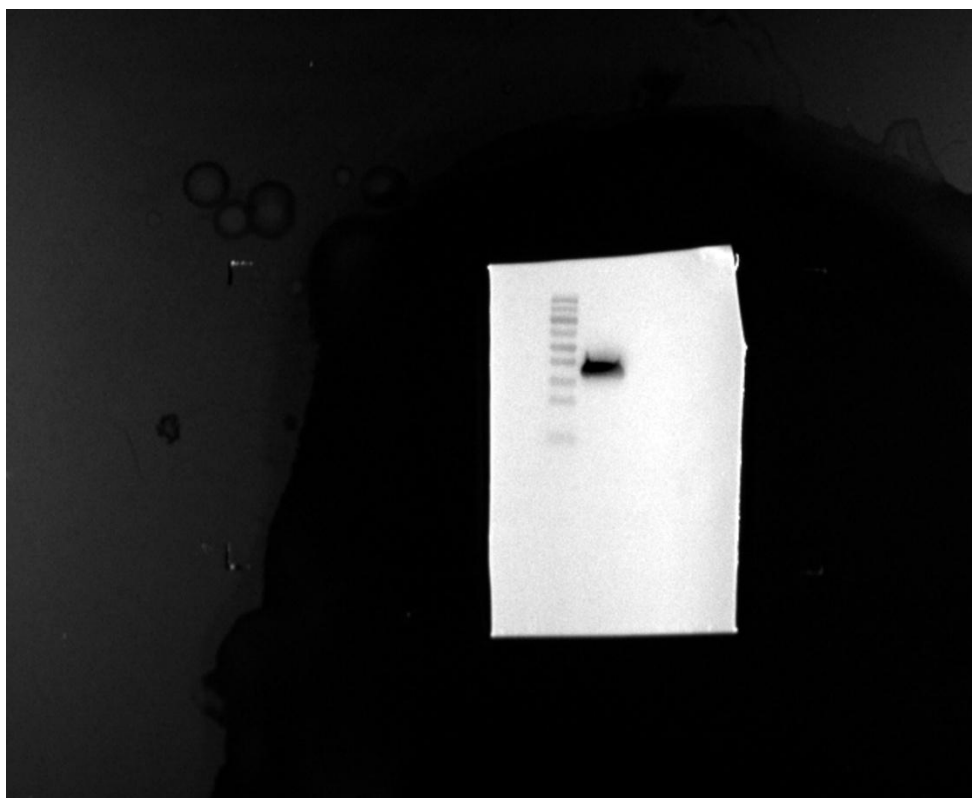

Figure S1: The original uncropped Western blot figures.

Supplement: Supplementary file 1 [file biology-15-00140-s001.zip › biology-4039187-supplementary.pdf]
